# Ciência em movimento: a epidemia do vírus Zika e as possibilidades de uma ciência mais responsiva

**DOI:** 10.1590/0102-311XPT135425

**Published:** 2025-11-10

**Authors:** Luciana Santos

**Affiliations:** 1 Pesquisadora independente, Brasília, Brasil.

Para Isabelle Stengers, filósofa da ciência, “*uma ciência triste é aquela em que não se dança*” ^1^ (p. 162), ideia que remete a uma ciência que não envolve engajamentos e paixões, e que não se deixa afetar pelas incertezas que os momentos de crise suscitam, caso das emergências em saúde. Trata-se, portanto, de uma ciência aberta a improvisações, ponderações e invenções, e que se propõe a experimentar, no laboratório e no campo, como aquela que se observa ao longo do livro *Misturas: Histórias de Pesquisas sobre o Vírus Zika* ^2^, organizado pelas antropólogas Thais Valim e Soraya Fleischer.

De início, é importante mencionar que o livro é parte de um conjunto significativo de publicações que buscou refletir sobre o surgimento da epidemia da síndrome congênita do Zika vírus (SCZ) em Recife (Pernambuco, Brasil), entre 2015 e 2016, quando ocorre o primeiro surto da doença. Nessa direção, o livro tem o mérito de apresentar ao leitor um dos múltiplos desdobramentos e resultados das pesquisas realizadas no âmbito do projeto *Uma Antropologia da Ciência do Zika: Resultados, Retornos e Epistemologias*, coordenado pelas organizadoras do livro.

Isso permite entrever um trabalho de fôlego que revela um esforço coletivo de uma equipe de pesquisadoras composta por alunas de graduação e pós-graduação em antropologia orientadas por Fleischer. Os rendimentos desse trabalho podem ser acompanhados em uma série de artigos publicados sobre o tema, bem como em teses de doutorado, dissertações de mestrado e trabalhos de iniciação científica. Note-se a preocupação da antropóloga e professora da Universidade de Brasília (UnB) com a formação e o treinamento de suas alunas. Nesse aspecto, a obra apresenta ressonâncias com publicações anteriores de Fleischer e que versam sobre questões de natureza epistemológica, metodológica, analítica, ética e política, e que singularizam sua trajetória como antropóloga, docente e pesquisadora. 

Ao seguirem a trilha desenhada pelos filósofos Latour & Woolgar ^3^, que consiste em problematizar a produção social do objeto científico, as organizadoras anunciam o objetivo da obra: mostrar como “*na fabricação da ciência, conteúdo científico e conteúdo social nunca estiveram separados*” ^2^ (p. 11). Entretanto, o que se destaca não é uma etnografia de laboratório nos moldes daquela realizada por Latour & Woolgar ^3^ no trabalho citado por Valim & Fleischer ^2^, mas um conjunto de 17 entrevistas, precedidas de uma apresentação, em que cientistas e profissionais de diferentes áreas do conhecimento expõem suas reflexões derivadas das experiências de pesquisa e clínica com a epidemia do vírus Zika no momento da sua emergência. Assim, ainda que não totalmente inédita nas ciências sociais, trata-se de uma forma de experimentação que se revela bastante interessante e original nos estudos de ciência e tecnologia. 

A partir de um esforço de rememoração dos interpelados, podem-se acompanhar, nos interstícios da produção do conhecimento científico, os entrelaçamentos ou misturas que se opõem à vocação universalista e à inquestionável objetividade da ciência moderna. Enquanto um exercício de reflexão posterior ao evento - as entrevistas foram realizadas quase uma década após o primeiro surto do Zika -, o que se apresenta são narrativas e histórias sobre as incertezas, os dilemas e os dramas que acompanharam a realização de pesquisa e assistência envolvendo crianças com deficiências graves e as dificuldades e dúvidas enfrentadas por suas mães e famílias. As entrevistas se movem, a princípio, em torno do que os profissionais caracterizam por um “susto” que a novidade implica e que se traduz no total desconhecimento da etiologia da doença. A seguir, desenvolvem-se por meio de narrativas que ligam problemas de financiamento de pesquisas, políticas e gestão públicas, questões de ordem pessoal e profissional, política universitária, entre outras. 

A escolha por iniciar as entrevistas com uma pergunta sobre a identidade de gênero e racial dos interlocutores permite - ainda que algumas vezes ignorada ou tratada de forma protocolar - que as histórias sejam enredadas a partir de elementos de vida particulares. A esse respeito, são relevantes os depoimentos de cientistas mulheres e negras que tiveram suas trajetórias profissionais atravessadas por questões identitárias. Observa-se, assim, a construção de diferentes camadas e matizes que dão maior densidade às histórias do vírus Zika, que, por sua vez, reforçam, entre outros aspectos, as preocupações e o comprometimento de Fleischer com temas prementes dos estudos de ciência e tecnologia no Brasil, caso do papel das mulheres na academia e nos espaços de produção do conhecimento científico.

O enredo da epidemia desvelado por cientistas de diferentes áreas evidencia os dilemas que cercaram os objetivos da pesquisa e o trabalho diário de assistência. Ao serem afetados pelo evento do Zika, esses cientistas realizaram mudanças nos protocolos de investigação científica, caso do tratamento dirigido a crianças saudáveis de mães infectadas pelo vírus Zika durante a gravidez, e que foram objeto das pesquisas do tipo caso-controle. Preocupações semelhantes aparecem no modo como os profissionais de campo ponderaram sobre o uso excessivo de recursos diagnósticos invasivos a que foram submetidas as crianças afetadas e que revelam uma rotina exaustiva para crianças e mães. Refletem também na escolha de alguns pesquisadores por uma ciência mais responsiva, o que significou assumir um posicionamento ético e político, e a responsabilidade pelos efeitos do conhecimento produzido com pessoas em condições de vulnerabilidade extrema. São exemplos desse tipo de mudança na condução das pesquisas as propostas de “devolutiva”, de “investigações em coautoria” com os envolvidos e o modo como os profissionais aventaram possibilidades de se realizar uma “tradução” dos resultados das investigações em termos mais acessíveis para mães e familiares. 

Ao longo das entrevistas surgem ainda problemas concernentes à captação de recursos para financiamento das investigações e desafios frente à articulação de redes de pesquisa que pudessem extrapolar o âmbito local. Aqui, pondera-se, sobretudo, sobre as históricas desigualdades regionais e o peso da temporalidade própria da investigação científica, que nem sempre acompanham as urgências e os dramas humanos suscitados por ocasião de um evento extraordinário. Em um primeiro momento, observa-se como o Zika permite atrair o financiamento e a atenção tanto do governo brasileiro quanto de entidades internacionais, a exemplo das redes de pesquisa que se desenharam a partir de parcerias com centros de investigação internacionais, além dos consórcios científicos que se estabeleceram entre diferentes regiões do país. Contudo, à medida que os casos de microcefalia se estabilizaram, os recursos ficaram cada vez mais escassos, seguindo de perto o deslocamento e o arrefecimento do interesse da mídia, das instituições governamentais e das agências financiadoras, ou mesmo de pesquisadores que identificaram no surto do Zika uma oportunidade para alavancar suas carreiras.

Junto a preocupações que se desenham no trânsito de profissionais entre o laboratório e a assistência, emergem outras questões relacionadas às condições de produção das investigações científicas sobre a nova síndrome, a exemplo dos desafios concernentes ao campo de divulgação científica. Ao adentrarem em discussões desse tipo, as histórias do livro permitem estabelecer paralelos com as práticas de produção de conhecimento próprias da área de estudos da antropologia. Acerca disso, interroga-se sobre o ofício do antropólogo no que diz respeito às relações que ele estabelece com os seus interlocutores, à realização de entrevistas, à experiência de campo e aos processos de escrita, publicação e divulgação de artigos acadêmicos. Com efeito, a obra é valiosa não apenas por oferecer importantes aportes a estudantes, acadêmicos e profissionais interessados na área restrita dos estudos de ciência e tecnologia, mas pelo potencial de inspirar reflexões sobre formas não convencionais de produção de conhecimento na antropologia e modos de conferir sentidos alternativos à produção científica. Para além disso, as histórias do Zika revelam como as epidemias são lugares privilegiados para observar uma ciência em movimento, que dança.
